# Supplementary material for: Maternal Iron Deficiency and Environmental Lead (Pb) Exposure Alter the Predictive Value of Blood Pb Levels on Brain Pb Burden in the Offspring in a Dietary Mouse Model: An Important Consideration for Cumulative Risk in Development
Source: Nutrients. 2023 Sep 22;15(19):4101. doi: 10.3390/nu15194101 (PMC10574741; doi:10.3390/nu15194101)
Supplement: Supplementary file 1 [file nutrients-15-04101-s001.zip › nutrients-2615358-supplementary.pdf]

Supplementary Table: Contributions of each dam and pup to each treatment and outcome measure

Group (# Dams)	Dam ID	Litter Size	# Females	# Males	HESKA		Dam Metals		Pup Metals		
					Dam	Pup	Blood	Femur	Blood	Femur	Brain
0-IN (n=14)	1	9	5	4	-	-	┼	┼	4	-	4
	2	13	9	4	-	-	-	-	4	-	4
	3	13	6	7	-	-	┼	┼	4	-	4
	4	5	2	3	-	-	┼	┼	2	-	2
	5	8	1	7	-	-	-	-	3	3	2
	6	8	4	4	-	-	-	-	4	4	2
	7	7	3	4	-	-	-	-	-	2	-
	8	7	3	4	-	-	-	-	-	2	-
	43	8	3	5	┼	8	-	-	-	-	-
	44	12	5	7	┼	12	-	-	-	-	-
	45	9	2	7	┼	9	-	-	-	-	-
	49	10	1	9	-	3	-	-	-	-	-
	50	8	4	4	-	4	-	-	-	-	-
	51	6	2	4	-	1	-	-	-	-	-
0-ID (n=12)	9	12	5	7	-	-	┼	┼	4	-	4
	10	9	6	3	-	-	┼	┼	4	-	4
	11	10	4	6	-	-	┼	┼	4	-	4
	12	13	7	6	-	-	┼	┼	2	-	2
	13	10	6	4	-	-	-	-	4	4	2
	14	12	6	6	-	-	-	-	4	4	2
	15	12	7	5	-	-	-	-	-	3	-
	16	10	8	2	-	-	┼	┼	-	2	-
	46	6	1	5	┼	6	-	-	-	-	-
	47	8	4	4	┼	5	-	-	-	-	-
	48	10	3	6	┼	-	-	-	-	-	-
	52	9	6	3	-	3	-	-	-	-	-
19-IN (n=4)	32	10	7	3	┼	4	┼	┼	**4**	4	**4**
	33	10	6	4	┼	4	┼	┼	**4**	4	**4**
	34	10	6	4	┼	4	┼	┼	**4**	4	**4**
	35	15	6	9	┼	7	┼	┼	**4**	4	**4**
19-ID (n=4)	36	9	5	4	┼	4	┼	┼	**4**	4	**4**
	37	6	3	3	┼	2	┼	┼	**4**	4	**4**
	38	11	9	2	┼	4	┼	┼	**4**	4	**4**
	39	12	3	9	┼	4	┼	┼	**4**	4	**4**
50-IN (n=11)	17	12	6	6	┼	4	-	-	-	-	4
	18	10	7	3	┼	4	-	-	-	-	4
	19	8	6	2	┼	4	-	-	-	-	4
	20	5	0	5	┼	2	┼	┼	2	3	2
	21	9	3	6	-	-	┼	┼	2	-	-
	22	11	4	7	-	-	-	-	4	-	-
	23	7	2	5	-	-	-	-	4	-	-
	24	7	4	3	┼	2	┼	┼	4	4	-
	25	5	1	4	-	-	-	-	-	2	-
	40	9	3	6	-	-	-	-	4	4	-
	41	11	6	5	-	-	-	-	2	1	-
50-ID (n=7)	26	9	2	7	┼	4	-	-	-	-	4
	27	9	4	5	┼	4	-	-	-	-	4
	28	3	2	1	┼	2	┼	┼	1	-	2
	29	12	9	3	┼	5	┼	┼	4	6	4
	30	5	2	3	-	-	┼	┼	2	-	-
	31	9	3	6	┼	3	┼	┼	4	4	-
	42	13	10	3	-	4	-	-	3	4	-

Symbols and Abbreviations: "┼" = Dam included in analysis column; Pups in "Bold" were used for Fig. 9
